# The Supersymmetry Genetic Code Table and Quadruplet Symmetries of DNA Molecules Are Unchangeable and Synchronized with Codon-Free Energy Mapping during Evolution

**DOI:** 10.3390/genes14122200

**Published:** 2023-12-12

**Authors:** Marija Rosandić, Vladimir Paar

**Affiliations:** 1 Department of Internal Medicine, University Hospital Centre Zagreb (Ret.), 10000 Zagreb, Croatia; 2 Croatian Academy of Sciences and Arts, 10000 Zagreb, Croatia; paar@hazu.hr; 3 Department of Physics, Faculty of Science, University of Zagreb, 10000 Zagreb, Croatia

**Keywords:** genetic code symmetry, mitochondrial genetic code, energy code, DNA quadruplet symmetry, classification of trinucleotides/codons

## Abstract

The Supersymmetry Genetic code (SSyGC) table is based on five physicochemical symmetries: (1) double mirror symmetry on the principle of the horizontal and vertical mirror symmetry axis between all bases (purines [A, G) and pyrimidines (U, C)] and (2) of bases in the form of codons; (3) direct–complement like codon/anticodon symmetry in the sixteen alternating boxes of the genetic code columns; (4) A + T-rich and C + G-rich alternate codons in the same row between both columns of the genetic code; (5) the same position between divided and undivided codon boxes in relation to horizontal mirror symmetry axis. The SSyGC table has a unique physicochemical purine–pyrimidine symmetry net which is as the core symmetry common for all, with more than thirty different nuclear and mitochondrial genetic codes. This net is present in the SSyGC table of all RNA and DNA living species. None of these symmetries are present in the Standard Genetic Code (SGC) table which is constructed on the alphabetic horizontal and vertical U-C-A-G order of bases. Here, we show that the free energy value of each codon incorporated as fundamentally mapping the “energy code” in the SSyGC table is compatible with mirror symmetry. On the other hand, in the SGC table, the same free energy values of codons are dispersed and a mirror symmetry between them is not recognizable. At the same time, the mirror symmetry of the SSyGC table and the DNA quadruplets together with our classification of codons/trinucleotides are perfectly imbedded in the mirror symmetry energy mapping of codons/trinucleotides and point out in favor of maintaining the integrity of the genetic code and DNA genome. We also argue that physicochemical symmetries of the SSyGC table in the manner of the purine–pyrimidine symmetry net, the quadruplet symmetry of DNA molecule, and the free energy of codons have remined unchanged during all of evolution. The unchangeable and universal symmetry properties of the genetic code, DNA molecules, and the energy code are decreasing disorder between codons/trinucleotides and shed a new light on evolution. Diversity in all living species on Earth is broad, but the symmetries of the Supersymmetry Genetic Code as the code of life and the DNA quadruplets related to the “energy code” are unique, unchangeable, and have the power of natural laws.

## 1. Introduction

Symmetries in nature are the main routes for restricting disorder as entropy increases, which enables evolution and the existence of living species. At the beginning of the 20th century, A. Einstein’s great advance was to put symmetry first—to regard the symmetry principle as the primary feature of nature that constrains dynamical laws. In relation to energy, he said “Everything is energy and that is all there is to it”. At the same time, Emmy Nether proved her theorem relating to symmetry and conservation laws [1]. E. Schrödinger in 1943 proposed that hereditary material must take the form of an “aperiodic crystal”, implying the presence of symmetries in the structure of DNA [2]. J. Monod in 1978 attached great significance to symmetry in biology, which must not be understood in purely geometrical connotations, but rather in a much wider sense, identical to that of order within a structure [3]. D. J. Gross 1996 postulated that symmetry principles dictate the form of the laws of nature. He concluded that, when searching for new and more fundamental laws of nature, we should search for new symmetries [4].

Non-random structures at different scales indicate a complex genome or genetic code organization. For example, the first and second Chargaff’s parity rules, which appear universally over almost all extant DNA genomes [5]. Also, symmetry was one of the key ingredients leading to the very strong organized our Supersymmetry Genetic Code structure on the base of the physicochemical laws in the form of the purine–pyrimidine symmetry net and codon–anticodon structures, with dominant double mirror symmetry within bases and codons [6,7].

Our scientific challenge was to interpolate the free energy values of codons/trinucleotides [8,9] in our SSyGC table and in all 10 A + T-rich and 10 C + G-rich quadruplets of DNA molecules [6,7] characterized by a purine–pyrimidine symmetry as well as the mirror symmetry, and to show how symmetries in the genetic code and DNA quadruplets directly influenced the relationship between codons/trinucleotides and their free energy.

## 2. Material and Methods

### 2.1. Characteristic of the SSyGC Table

**Figure 1:**
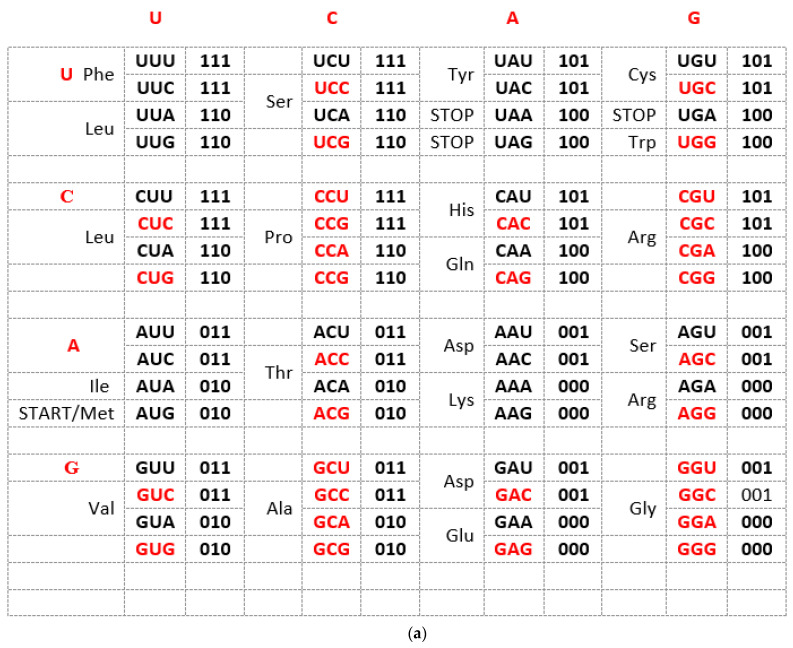

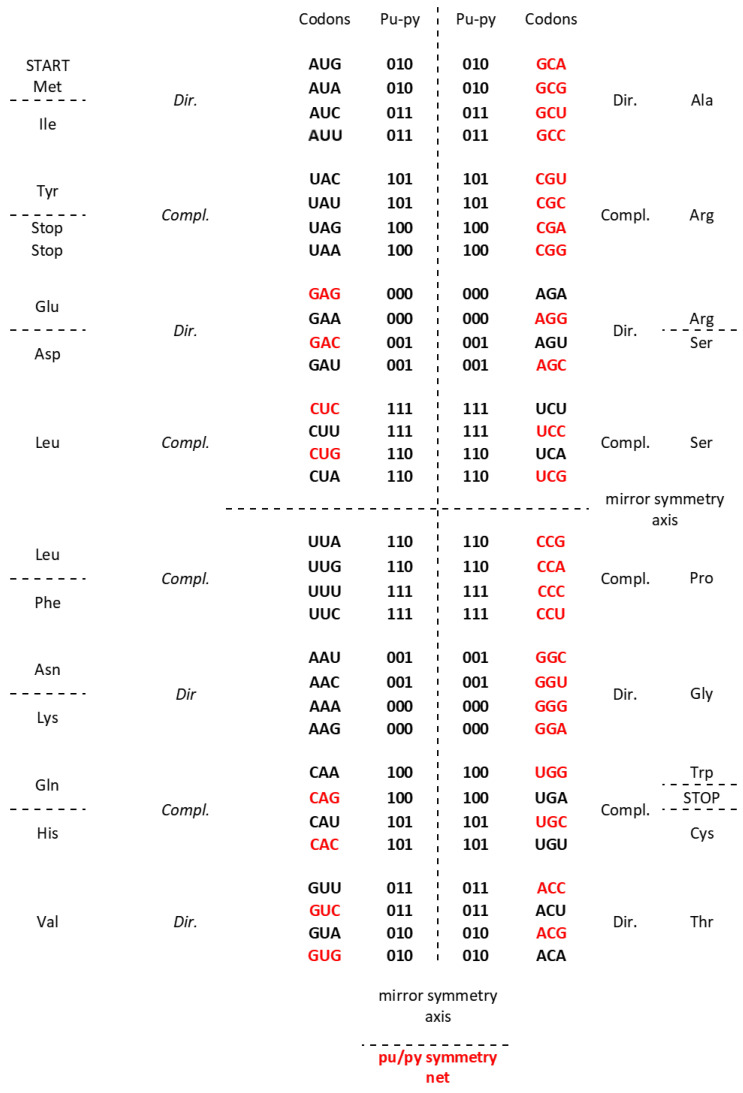

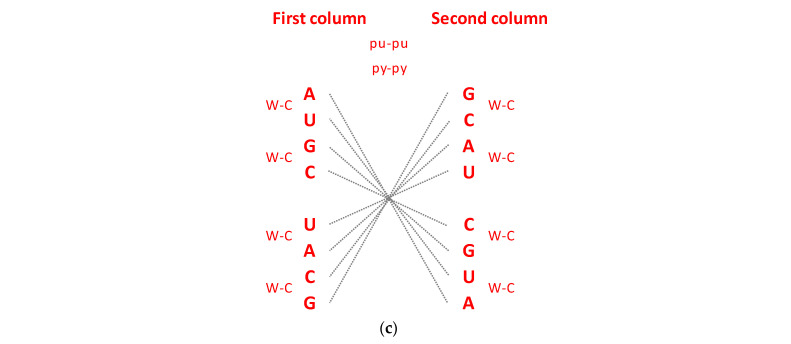
(**a**) The Standard Genetic Code table is structured horizontally and vertically on the alphabetically U-C-A-G-based manner and suffers from an inability to show the complete physicochemical symmetry between codons. There is only partial mirror symmetry between purines (0) and pyrimidines (1) in the first and second columns as well as in the third and fourth columns. A + U-rich (black) and C + G-rich (red) codons are not in an alternate range regularity in each row. There is not direct and complement (codon 5′3′ ↔ anticodon 3′5′) altering ranging of the codon boxes. (**b**) The Supersymmetry Genetic Code table with double mirror symmetry and horizontal and vertical mirror symmetry axis. There are, in the same row, A + U-rich and C + G-rich alternate codons between two columns. Both columns have the same distribution of purine–pyrimidine profile; simultaneously, the same profile distribution pairs of codon rows within each box. There is a purine–pyrimidine symmetry between the bases and codons, and direct–complement symmetry (codon 5′3′ ↔ anticodon 3′5′ as well as Watson–Crick pairing) of codons between direct and complement codon boxes. In such a way, for the first time, the sextets for Serine, Arginine, and Leucine, each with six codons, are positioned in continuity. It is interesting that the AUG start signal is at the beginning of the Supersymmetry Genetic Code table. It is important that the purine–pyrimidine symmetry net is in a central position in the code as the “golden rule”; this is common for all RNA and DNA species and unchangeable during evolution. Note: 0 pu—purine; 1 py—pyrimidine; black—A + U-rich codons; red—C + G-rich codons. (**c**) The center point mirror symmetry for the first base in the whole SSyGC table. First base in each box from the left and right column of the SSyGC table. The 1st, 2nd, 3rd, 4th, 5th, 6th, 7th, and 8th base in the left column correspond to the 8th, 7th, 6th, 5th, 4th, 3rd, 2nd, and 1st base, respectively; all connecting lines pass through the center point. Each base represents a whole box because the first and second bases of the four codons in each box of code are identical.

The Standard Genetic Code (SGC) table included in all biology and genetics textbooks and all other known genetic code tables structured horizontally and vertically on the alphabetically U-C-A-G base manner suffer from an inability to show the complete physicochemical symmetry between codons. (Figure 1a). Our novel SSyGC table follows the stereochemical theory, which postulates that the structure of the code is determined by a physicochemical affinity between amino acids and codons or anticodons [10,11,12]. The SSyGC table (Figure 1b) encompasses five symmetries: (1) double mirror symmetry between all purines and pyrimidines on the basis of vertical and horizontal mirror symmetry axis created purine–pyrimidine symmetry net, as well as the center point mirror symmetry (Figure 1c) for the first base of all codons (in each of 16 boxes first and second base are identical) in the SSyGC table; (2) purine–pyrimidine symmetry on the basis of Watson–Crick pairing in the alternately boxes of genetic code; (3) direct–complement symmetry on the basis of Watson–Crick pairing between codons in the alternately boxes of genetic code; (4) A + T-rich and C + G-rich symmetry between alternating codons in the same row of both columns of code (Figure 1b); (5) symmetry between the position of amino acids [6,7]. Constructed from two columns with eight boxes which are made up of four codons each, the SSyGC table is more physiological. Namely, with the horizontal transformation through a purine–purine, as well as a pyrimidine–pyrimidine exchange of bases in codons, A + T-rich and C + G-rich codons alter between the two columns in the same row through the entire genetic code (Figure 1b). In contrast, with vertical alteration in direct and complement (codon 5′3′ ↔ anticodon 3′5′) pairs of boxes, we obtained Watson–Crick pairings between the bases and codons in the entire genetic code (Figure 1b).

In addition, with our novel configuration of the two-columns of the genetic code table, we also found mirror symmetry between all purines and pyrimidines as well as between the second and third bases of all codons. At the same time, the mirror pairing of the first base of codons in all boxes alternate over both purines (A ↔ G), and both pyrimidines (U ↔ C). We found also that the first base of all codons in the SSyGC table has the center-point mirror symmetry (Figure 1c).

The novel result is that the SSyGC table deduces codons on the binary code with A, G purines as “0” and U, C pyrimidines as “1”, and has the unique physicochemical purine–pyrimidine symmetry net with double vertical and horizontal mirror symmetry (Figure 1b). The purine–pyrimidine symmetry net is common for all RNA and DNA living species. It is also common for all more than thirty nuclear and mitochondrial genetic codes. It is important to point out that the purine–pyrimidine symmetry net has remained unchanged during all of evolution [6,7].

We show that the purine–pyrimidine symmetry net enables an automatic transformation of the SSyGC table into two-fold DNA strand sequences with the Watson–Crick pairing (A ↔ U, C ↔ G), which appears by linearizing codons from direct boxes for the top strand and the complement boxes for the bottom strand. This is an analogous form to the 5′3′ codon and the 3′5′ anticodon [6,7].

The SSyGC table also has a symmetrical arrangement of codons and amino acids. For the first time, we can see that our SSyGC table contains three codon sextets for Serine, Arginine, and Leucine, each with its codons positioned vertically and in continuity. At the same time, the position of the start signal AUG is symbolically at the top of the first column of the SSyGC table. The codons of the three stop signals (UAA, UAG, and UGA) have a mutual diagonal mirror symmetry position [6,7].

In conclusion, we can say that the SSyGC table has five symmetries: purine–pyrimidine symmetry, double mirror symmetry, A + T-rich and C + G-rich symmetry, direct–complement symmetry, and symmetry between amino acids with the same position between divided and undivided codon boxes in relation to horizontal mirror symmetry axes. The SGC table has none of these symmetries [13].

### 2.2. Characteristic of DNA Quadruplet Symmetries

**Figure 2:**
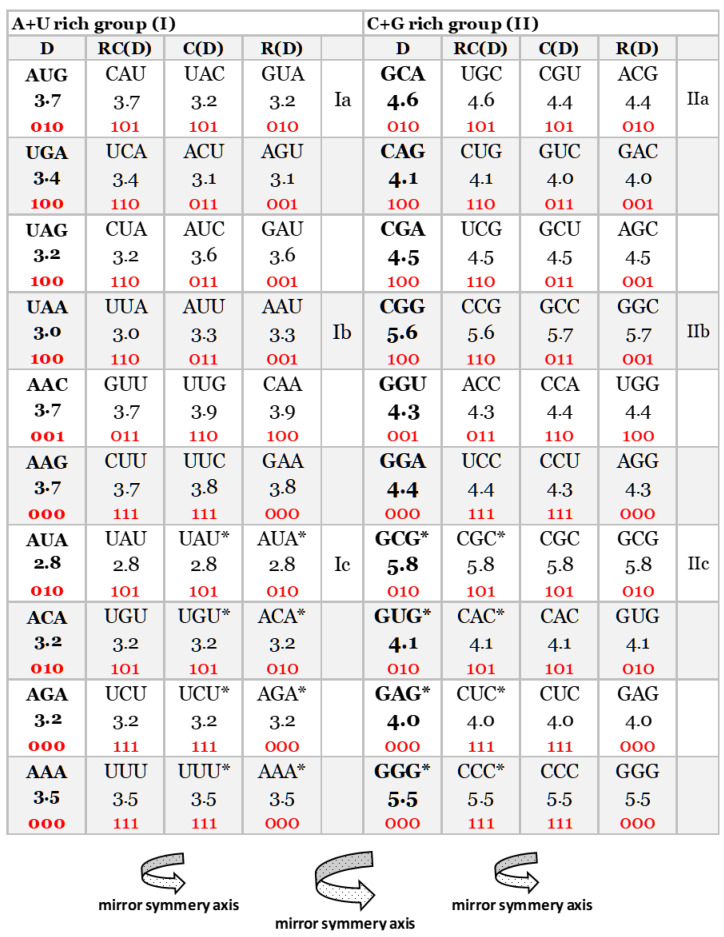
Our quadruplet classification of 61 codons and 3 stop signals (with U-uracil) for genetic code, or trinucleotides (with T-thymine instead of uracil) for RNA and DNA genomes with incorporated the free energy of codons (reading unidirectionally from 5′ to 3′). Each quadruplet is unique and consists of codons or trinucleotides denoted as direct D, and reverse complement from direct RC(D), complement from direct C(D), and reverse from direct R(D). The ten A + U-rich (group I) and ten C + G-rich (group II) quadruplets are organized in three subgroups. The Ia subgroup consists of nonsymmetrical codons/trinucleotides containing four different nucleotides; the Ib subgroup consists of nonsymmetrical codons/trinucleotides containing two different nucleotides; the Ic subgroup consists of symmetrical codons/trinucleotides which contain duplicated codons/trinucleotides labeled with an asterisk (D = RC, C = R). The first four A + U-rich quadruplets we generated with start/stop signals: AUG, UGA, UAG, and UAA. The C + G-rich trinucleotides correspond to purine–purine and pyrimidine–pyrimidine transformations of A + U-rich codons/trinucleotides. Three symmetries are present in our codon/trinucleotide classification: (1) purine–pyrimidine symmetries in each quadruplet; (2) purine–pyrimidine symmetries within and between A + U-rich and C + G-rich quadruplets in the same row of the classification; (3) mirror symmetry between direct–reverse and complement–reverse complement in the same quadruplet. For clarity, the white and grey rows are alternating, to emphasize pairs of A + T-rich and C + G-rich codons. Note: 0—purine; 1—pyrimidine. Namely, it is irrelevant which codon/trinucleotide in the quadruplet is direct, because the other three are accordingly adapted.

**Figure 3:**
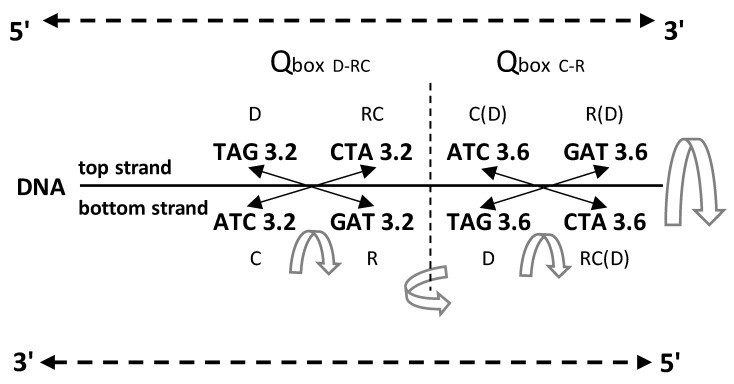
An example of symmetries in one A + T-rich quadruplet from the DNA molecule with the incorporated free energy of trinucleotides. The free energy value follows quadruplet symmetries in bidirectional form (top strand 5′3′, bottom strand 3′5′). There are three quadruplet symmetries: purine–pyrimidine symmetry, direct–complement symmetry, both on the principle of Watson–Crick pairing between DNA strands, and important mirror symmetry between both DNA strands in Qbox_D-RC_ as well as Qbox_c–R_, and between both boxes. Their mirror symmetry with complementary base pairing leads directly to Chargaff’s second parity rule (see the text). The free energy of trinucleotides follows mirror symmetry. The same symmetries exist in the SSyGC table. D—direct; RC—reverse complement; C—complement; R—reverse; C(D)—complement from direct of four trinucleotides; R(D)—reverse from direct; RC(D)—reverse complement from direct; 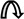 mirror symmetry. The inputted measured free energy values are from [8,9].

An important characteristic of the SSyGC table is the direct transformation of genetic code in the DNA molecule through linearizing codons from a direct pair of codon boxes for the top strand alternately, with codons from a complement pair of boxes for the bottom strand of DNA. In this case, the alignment of the 192 bases (61 codons and 3 stop signals) contains 10 A + T-rich quadruplets and 10 C + G-rich quadruplets (Figure 2) [7]. Each quadruplet contains four types of trinucleotides: direct, reverse complement, complement, and reverse. We relate Chargaff’s second parity rule to the interstrand mirror symmetry in 10 A + T-rich and 10 C + G-rich symbolic purine–pyrimidine symmetry quadruplets of trinucleotides mapped to the double-stranded genome. The symmetries of the Q-box corresponding to quadruplets can be obtained due to a combination of Watson–Crick base pairing and Chargaff’s second parity rule. We show that, alternatively, assuming the natural symmetry law for DNA creation and conservation that each trinucleotide (or mono/oligonucleotide) in one strand of DNA must also simultaneously appear in the opposite strand, regardless of localization, automatically leads to direct–reverse mirror symmetry between both strands of the Q-box. In conjunction with Watson–Crick base pairing, this also automatically generates 5′3′ ↔ 3′5′ bidirectional strand construction of the DNA molecule and, automatically, Chargaff’s second parity rule [5,7]. The quadruplet symmetries are characterized by the same symmetries as the SSyGC table: purine–pyrimidine symmetry and direct–complement symmetry. These are based on the trinucleotides between both strands and on the principle of the Watson–Crick pairing, and the mirror symmetry within each Q-box and between Q-box_D-RC_ and Q-box_C—R_. (Figure 3). Empirically, it is known that the relative frequencies of all four trinucleotides within each Q-box are almost equal, although they do differ among both Q-boxes. However, the sum of relative frequencies from both strand of each member of quadruplets is precisely the same [5,7].

### 2.3. Free Energy Values Incorporated in the SSyGC Table and in DNA Quadruplets

**Figure 4:**
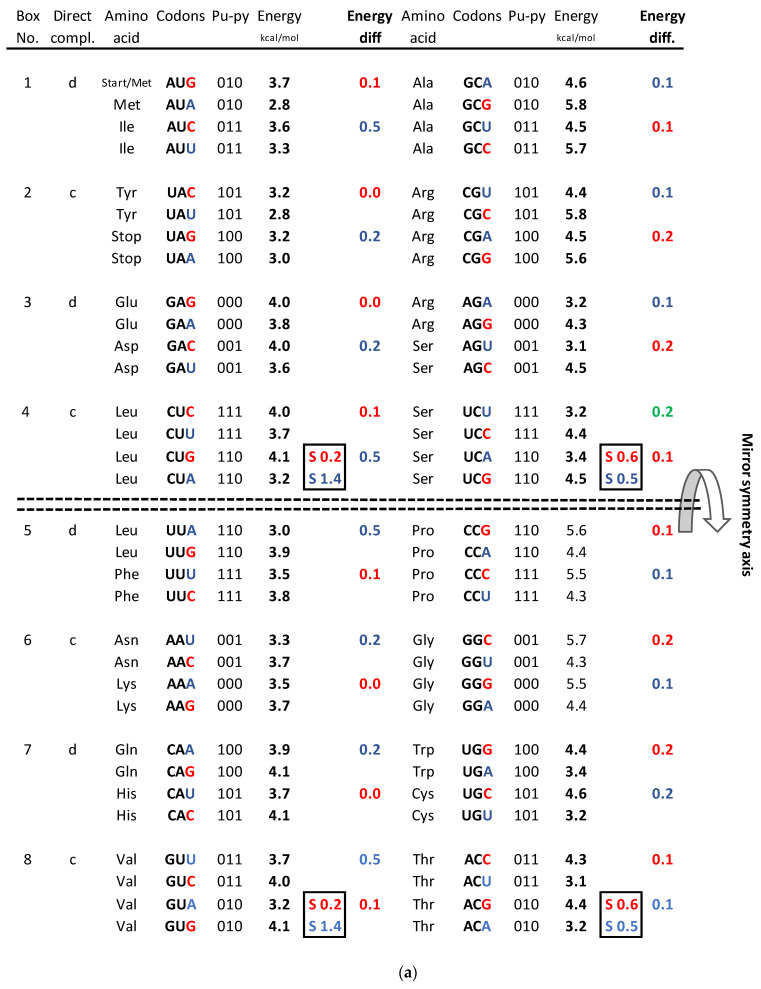

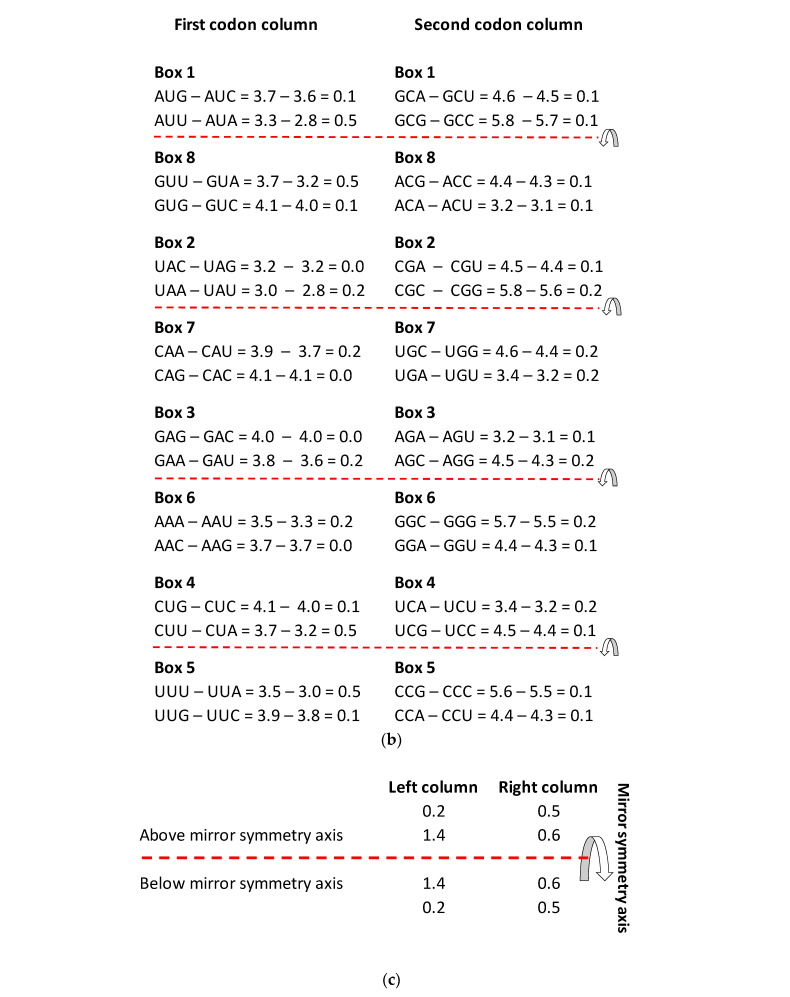

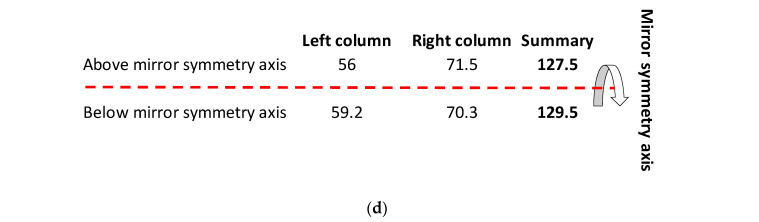
(**a**) The energy code incorporated in the SSyGC table, which is adjusted for the mitochondrial human genetic code. Placing the axis of horizontal mirror symmetry in the central position of the SSyGC table, we observed an excellent agreement of the sum free energy of codons between both halves of code (see (**d**)) and complete symmetry for **differences** between the free energy of codons in each box which has at the third position C and G (strong) or A end U (weak) nucleotides. Therefore, in the SSyGC table, we see symmetries between differences in codon-free energy in the boxes 1 and 5, 2 and 6, 3 and 7, and 4 and 8, with a slight deviation in the second column. Namely, each box contains four codons with the same first and second bases, and the decisive free energy difference depends only on the third base in each codon. Within each box in the SSyGC table, the codons with G and C third strong base have larger values of free energy than codons with A and U weak third base (see text). Pu—purine; py—pyrimidine; red—codons with C and G third base; blue—codons with A end U third base; S sum. The inputted measured free energy values are from [8,9]. (**b**) Differences in free energy codons between the A + U-rich weak codons and the C + G-rich strong codons from alternately boxes in both columns of the SSyGC table. There appears horizontal mirror symmetry between differences in codon-free energy in the horizontal pairs of boxes 1 and 8, 2 and 7, 3 and 6, and 4 and 5, with only a slight deviation in the second (right) column. (**c**) Sum of differences between free energies within each codon with A or U weak third base and codon with C or G strong third base in the same box. Only the third base of codons in each box is different and makes the difference in the free energy between their codons (all four codons in each box have first and second base identical). Energy differences are defined as in (**a**). Sums of energy differences show the symmetry with respect to the horizontal mirror symmetry axis of the SSyGC table (see (**a**)). (**d**) Sum of free energy of all codons in kcal/mol with respect to the horizontal mirror symmetry axis of the SSyGC table is almost identical above and below mirror symmetry axis (127.5 + 129.5 = 257). The difference of 2 between summary values is probable a technical mistake. This free energy mirror symmetry of codons is result of identical purine–pyrimidine symmetry net and identical number of A + T-rich and C + G-rich codons above and below horizontal symmetry axis of code.

We incorporated the free energy values of each codon in the SSyGC table adapted for the human mitochondrial genetic code. In this case, only the assignment of the amino acid for two codons is changed: AUA for Methionine instead of Isoleucine, and UGA of Tryptophane instead for the stop signal. This is the case in the nuclear human genetic code. It is very important to emphasize that the purine–pyrimidine symmetry net is completely identical in mitochondrial human genetic code to the SSyGC table (compare Figure 1b and Figure 4a).

**Figure 5:**
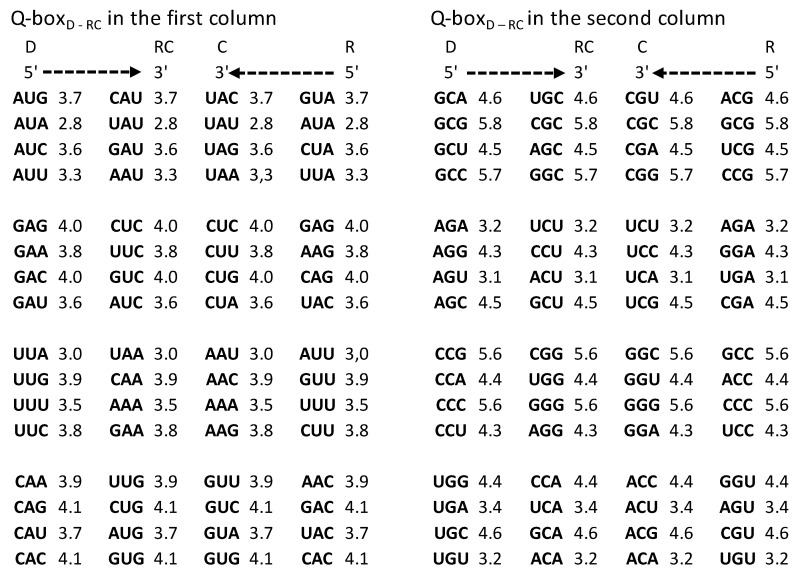
Quadruplet boxes (Q-boxes) of codons (D—direct; RC—reverse complement; C—complement; R—reverse) arranged as in the SSyGC table in the form of DNA quadruplets with mirror symmetry (see also Figure 2 and Figure 3). The corresponding value of free energy is given with each codon. It is necessary to read bases/codons of the Q-box bidirectionally (5′3′ top strand, 3′5′ bottom strand). Full agreement of the identical free energy values for all four members in each Q-box is present. The inputted measured free energy values are from [8,9].

We also incorporated the free energy values of each codon in the SSyGC table by creating DNA quadruplets (direct–reverse complement–complement–reverse) (Figure 4). We incorporated the free energy values of codons/trinucleotides directly in quadruplets as in the example in Figure 3. We also compared free energy between direct and reverse–complement trinucleotides and complement and reverse of A + T-rich and C + G-rich DNA quadruplets of our classification of trinucleotides/codons on the principle of strand symmetry (Chargaff’s second parity rule) (Figure 5).

The free energy values for all 61 codons/trinucleotides and 3 stop signals are downloaded from [8,9]. The value of the free energy of codons is expressed in kcal per mol. The authors used spectroscopic and calorimetric techniques to characterize the free energy of each codons/trinucleotide.

**Figure 6:**
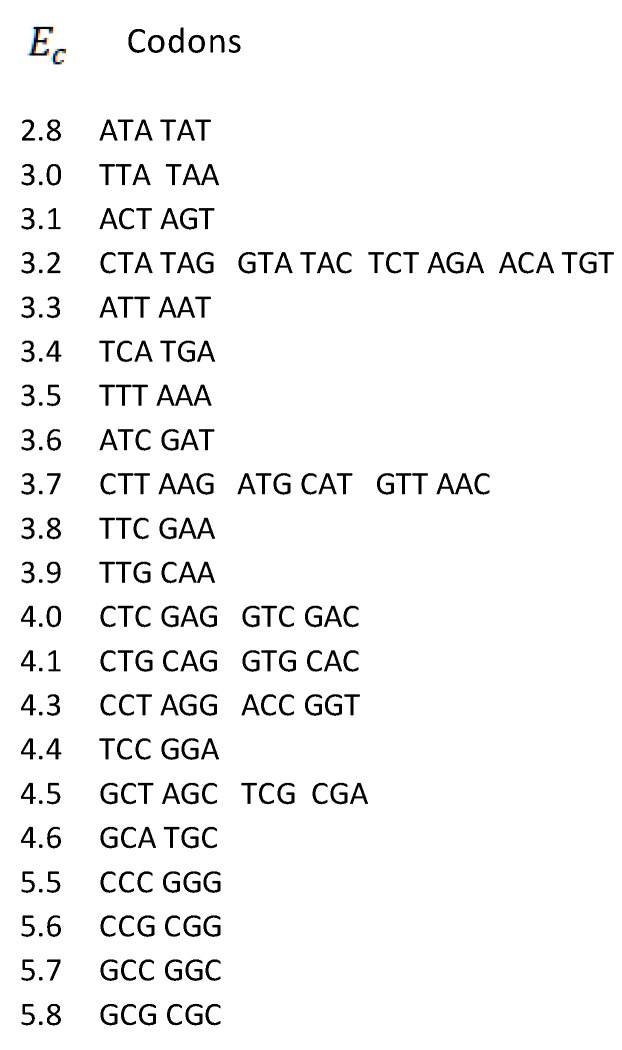
Degeneracies of free energies vs. codons for human mitochondrial DNA. Codons are ordered in pairs according to the strand symmetry (Chargaff’s second parity rule).

Measured values of free energy $E_{c\;}$ (kcal/mol) are taken from Breslauer et al. (1986) [9] and Klump et al. (2020) [8]. In [9], it was shown that the free energy of each codon is equal to the free energy of its reverse complement. These values were further inserted into the mitochondrial human genetic code table (Figure 1), where they were randomly scattered. Here, we first display the free energy values in the energy versus codon table. In most cases, to each energy corresponds only one codon pair, i.e., the twofold codon degeneracy. For example, to the energy 2.8 kcal/mol correspond the codon ATA and its reverse complement TAT, i.e., the twofold codon degeneracy. Additionally, there are four cases of fourfold codon degeneracy; for example, the energy 4.0 corresponds to four codons, CTC, GTC, and their reverse complements, GAG, GAC, respectively. There are two cases of sixfold degeneracy; for example, the energy 3.7 corresponds to three codons, CTT, ATG, GTT, and their reverse complements, AAG, CAT, AAC, respectively. The highest, eightfold degeneracy appears for the energy 3.2, corresponding to eight codons, CTA, GTA, TCT, ACA, and their reverse complements TAG, TAC, AGA, and TGT, respectively (Figure 6).

## 3. Results

We compared not only free energy values between codons and symmetries in the SSyGC table but also the **differences** in the free energy values between C + G-rich strong codons and A + T-rich weak codons, and their relationship between symmetries of the SSyGC table (Figure 4).

- In the first column, the summary of the free energy of all codons of the SSyGC table (A + U-rich 24 codons, C + G-rich 8 codons) is 115.2 kcal/mol (56 above and 59.2 below axis of mirror symmetry).
- In the second column, the summary of the free energy of all codons of the SSyGC table (A + U-rich 8 codons, C + G-rich 24 codons) is 141.8 kcal/mol (71.5 above and 70.3 below axis of mirror symmetry).
- The free energy of all codons of the SSyGC table is 257 kcal/mol.
- In the whole genetic code, the free energy of all 32 weak A + U-rich codons is 108.6 kcal/mol, and of all strong 32 C + G-rich codons 148.4 kcal/mol (27% higher).
- All 16 symmetrical codons in the direct–complement pair of boxes have completely identical free energy of codons (e.g., AUA 2.8 kcal/mol–UAU 2.8 kcal/mol; CCC 5.5 kcal/mol–GGG 5.5 kcal/mol) (Figure 2).
- The free energy difference between codons in each box which has the third base A and U or C and G in the upper and lower half of genetic code are completely identical, which coincide with the horizontal mirror symmetry axis of the SSyGC table (Figure 4b,c).
- The sum of the free energy of all codons in kcal/mol with respect to the horizontal mirror symmetry axis of the SSyGC table is almost identical above and below mirror symmetry axis (127.5:129.5) (Figure 4d). The minimal difference of 2 is probably a technical mistake.

We show that the value of the free energy depends on the strong G and C bases with 3 H-bonds and the week A and U bases with 2 H-bonds of codons. Namely, the free energy of strong C + G-rich codons is 27% higher than the weak A + U-rich codons. At the same time, the value of the free energy depends on the third base of all four codons in each box, because the first two bases are identical and there are no differences in the free energy between them (Figure 4a).

The SSyGC table has the double mirror symmetry on the principle of vertical and horizontal mirror symmetry axis between purine–pyrimidine codons. Vertical mirror symmetry axis separates the SSyGC table in two columns with the purine–pyrimidine mirror symmetry. However, the mirror symmetry of the codons free energy between columns do not exist. Namely, A + U-rich codons with lower free energy are dominant in the left column (A + U-rich 24, C + G-rich 8), and C + G-rich codons with higher free energy are dominant in the right column (C + G-rich 24, A + U-rich 8). In contrast, the horizontal mirror symmetry axis separates the SSyGC table in two horizontal halves which have the identical number of A + T-rich and C + G-rich codons. At this way, the sum of free energy between codons of both halves is almost identical and the horizontal mirror symmetry of the free energy is created (Figure 4d).

The fascinating identical summary between difference of the free energy codons in the upper and lower part of both columns as result of a horizontal mirror symmetry speaks in favor of energy balance and maintaining the integrity of the genetic code. The mirror symmetry of the free energy in the SSyGC table is seen due to the third base in the codons, whose role in the creation of symmetry in the Standard Genetic Code was previously not considered [13]. We cannot see this correlation directly in the Standard Genetic Code table. It has only a partial vertical mirror symmetry between purines and pyrimidines in first and second as well third and fourth column of genetic code and has not a horizontal mirror symmetry.

In the [8,9] the authors Breslauer et al. (1986) and Klump et al. (2020) discovered equality of the free energy of the trimeric duplexes formed by antiparallel complementary codons (synonym is direct–reverse-complement codons) independently from the Standard Genetic Code table structure. Our SSyGC table directly shows the mirror symmetry of the sum free energy values of codons as well as sum between difference of A + T-rich and between C + G-rich codons in the upper and lower part of code.

It should be stressed that the free energy values of trinucleotides in each quadruplet of DNA are also in agreement with Chargaff’s second parity rule (strand symmetry) (Figure 5). It is necessary to recognize that Chargaff’s second parity rule of DNA molecules automatically results from dominant quadruplet mirror symmetry (Figure 3). Each quadruplet contains an identical relative frequency of direct and revers complement of trinucleotides, as well as complement and reverse in both strands of the DNA genome [5] (Figure 3). However, Chargaff’s second parity rule does not recognize in the same strand complement and reverse equality of frequencies in the shape of Qbox_c-r_, which leads to the formation of DNA quadruplets between both strands (Figure 3).

Comparing the free energy of codons/trinucleotides as DNA quadruplet partners of the Q-boxes (Qbox direct–reverse complement and Qbox complement–reverse) we obtain their identical free energy (Figure 3, Figure 5 and Figure 6). Namely, because of the Q-box mirror symmetry with DNA bidirectional reading (5′3′ top strand, 3′5′ bottom strand), there are the same free energy values between all four members of each Q-box of each of twenty quadruplets. Such equality of the free energy of trinucleotides between both strands of DNA molecule on the ground of quadruplets mirror symmetry rise to integrity of large chromosomes like, for example, the human chromosome 1 with about 249 million bp.

## 4. Discussion

In the recently reported review about energy mapping of the genetic code, authors Horst H. Klump, Jens Völker, and Kenneth J. Breslauer [9] concluded that “the genetic code driven by differential codon stabilities, evolved the influence and regulation of a series of interlocking thermodynamics cycles”. Comparing the “energy code” with the Standard Genetic Code (SGC) table, he proceeded “that the genetic code table shows stabilizing free energy values for the trimeric duplexes formed by antiparallel complementary codons”. These stability parameters were calculated using spectroscopically and calorimetrically-derived free energy values [9] with the conclusion that “under the influence of the laws of thermodynamics, this “energy code” evolved into a nearly singular code across all living species”. The authors compared this “energy code” with the SGC table, which is an artificial construct of representing the empirical codon–amino acid assignments, based horizontally and vertically on the U-C-A-G ordering of nucleotides. This way of representation does not include natural symmetry of complementary codons on the principle of Watson–Crick purine–pyrimidine pairing and complete double vertical and horizontal mirror symmetry between bases and codons. Namely, all codons in the SGC table are dispersed with respect to the physicochemical complementary properties.

In another approach, Bashford, and coworkers in 1998 [14] presented an analysis of the supersymmetry structure and evolution of the genetic code in terms of classical superalgebras and have presented a model (but not a new genetic supersymmetry code with the complete code symmetries as our the SSyGC table) based on the decomposition of a family as 64-dimensional representation with exchange symmetries in codon quartets.

The standard genetic code (SGC) table was elaborated by listing the stabilizing free energy values for each fully paired codon/reverse complement codon duplex [9], calculated using previously reported calorimetrically derived free energy values. The mapping in terms of codon/reverse complement codon energetics was justified on multiple physicochemical levels [9,15,16,17,18]. Klump et al. [9] have shown that one can envision the genetic code as composed of interlocking thermodynamic cycles compared with the hypercube of eight cube octet that allow codons to “evolve” from each other through transition and transversion. He calculated empirically that 61 possible codons and three stop signals of genetic code have 32 pairs of trimeric complementary duplexes (synonym of direct ↔ reverse complement) and concluded that “the code has a twofold symmetry that is not apparent from the conventional Standard genetic code table but becomes apparent when the codon–anticodon energies are listed for each triplet.” Namely, during the time of writing their article, the SSyGC table was not known, as it was not published until 2022 [6]; therefore, it could be not compared to physicochemical symmetries like codon–anticodon and mirror symmetry of genetic code. Numerous variants of the genetic code table were created with the goal to discover symmetries, but solutions were always only partial.

We show that our SSyGC table is structured of five physicochemical symmetries and therefore is superior in relation to the SGC table, which has only alphabetic symmetry based on U-C-A-G ordering of bases. The SSyGC table is universal with its purine–pyrimidine symmetry net common for all RNA and DNA species and has remained unchanged during evolution. In scientific literature, it was estimated that there are 10^84^ alternative possibilities of different variations of genetic codes [19]. Many efforts were made in attempt to discover physicochemical symmetries between bases, codons, and amino acids into the single genetic code. Our unique SSyGC table discovers physicochemical symmetries between all these elements—purine–pyrimidine bases, direct and complement (5′3′ codons ↔ 3′5′ anticodons) pairing of bases and codons—regularly exchange A + T-rich and C + G-rich codons, and the corresponding symmetrical arrangement of amino acids. This cycle is fascinatingly closed by double vertical and horizontal mirror symmetry in which the free energy of codons is also embedded. Due to the requirements of this symmetry, the position of each codon is strictly defined in the SSGC table and cannot be replaced by another codon.

We have proved that the purine–pyrimidine symmetry net is common for all RNA and DNA living natural species and nuclear and mitochondrial genetic codes and has remained unchanged during evolution. There are more than thirty alternative genetic codes from the Standard Genetic Code table. However, in the SSyGC table, individual amino acids change only a codons number with capture a codon/codons usually from the neighboring amino acid [6,7], but the position of codons and the purine–pyrimidine symmetry net stay unchangeable. In such a way any newly discovered genetic code table will have completely the same purine–pyrimidine symmetry net as the SSyGC table.

We show that completely identical symmetries of genetic code and energy code also created all 10 A + T-rich and 10 C + G-rich quadruplets from our classification of trinucleotides in both strands of DNA molecule (Figure 5).

Intervention into *Escherichia coli* genetic code through laboratory experiments, changing the chemical structures of some amino acids or removing at most 2–3 codons, the remaining codons stay in the same symmetrical position within the purine–pyrimidine symmetry net, but none of them can replace a consequence of mutations. Namely, each codon has a strictly defined position within the SSyGC table. The result is violation symmetries of genetic code and of energy code; this does not preserve vitality or results in fatal outcomes, even for simple organisms such as *E. coli* [20,21].

Commenting on Darwin’s evolution theory, Horst H. Klump and coauthors [9] concluded: “Evolution of the code was influenced by differential energetics, as thermodynamics is the most general and universal branch of science that operates over all time and length scales.”. We show that the dominant double mirror symmetry with Watson–Crick pairing is the basic structure of the SSyGC table, DNA quadruplets, and classification of trinucleotides/codons. The symmetries corresponding to the SSyGC table are accompanied by identical symmetries in the form of DNA quadruplets. Both are perfectly imbedded in the energy mapping of codons/trinucleotides and represent three mutual, well-defined, harmonized, and unchangeable features during all of evolution. Diversity in all living species on Earth is broad, but the symmetries of the genetic code as the code of life and DNA quadruplets, both related to the energy code, are unique and have remained unchanged throughout evolution; as a consequence, they have the power of natural law.

## Data Availability

Data publicly available.
